# MEK nuclear localization promotes YAP stability via sequestering β-TrCP in KRAS mutant cancer cells

**DOI:** 10.1038/s41418-019-0309-6

**Published:** 2019-03-04

**Authors:** Huanji Xu, Sheng Zhou, Hongwei Xia, Huangfei Yu, Qiulin Tang, Feng Bi

**Affiliations:** Department of Abdominal Oncology, Cancer Center and Laboratory of Molecular Targeted Therapy in Oncology, West China Hospital, Sichuan University, Chengdu, Sichuan Province 610041 China

**Keywords:** Cell biology, Oncogenes

## Abstract

Tumours manage to survive the ablation of mutant KRAS, despite the development of KRAS-targeted drugs. Here we describe that inhibition of mutant KRAS promotes MEK nuclear localization as an alternative mechanism of KRAS-targeted drugs resistance. Tissue microarray analysis in colon tumours shows that aberrant MEK nuclear localization is closely related to YAP levels and tumour malignancy. MEK nuclear localization could sequester β-TrCP from cytoplasmic inactive YAP, then stabilizing YAP. Mutant KRAS restrains MEK within the cytoplasm via IQGAP1, inhibiting MEK nuclear translocation. Trametinib, an allosteric MEK inhibitor, could prevent MEK nuclear localization and subsequently promote YAP degradation. In vitro and in vivo results suggests that inhibition of MEK nuclear localization by trametinib synergizes with KRAS knockdown or deltarasin treatment in suppressing the viability of KRAS mutant colon cancer cells. Our study provides new insights into the mechanisms of resistance to KRAS ablation, and suggests novel strategies for the treatment of KRAS-mutant colon cancers.

## Introduction

Somatic mutations in KRAS are the most common activating lesions in human cancers, including pancreas, lung and colon cancers [1]. The multiple signalling pathways engaged by mutant KRAS form the foundation for its diverse biological roles in proliferation, survival, metabolism and tumour microenvironment remodelling [2]. In recent years, some promising inhibitors, such as ARS-853 [3], deltarasin [4], rigosertib [5], exosomes [6] and AZD4785 [7] have renewed hope for the development of KRAS inhibitors, seeming to change the previous perception that KRAS was undruggable. But growing evidence has shown that cancer cells manage to survive the ablation of mutant KRAS by re-activation of compensatory pathways, such as YAP [8, 9] and AKT [10, 11] in KRAS-dependent pancreatic cancer mouse models. These studies indicate that seeking methods to reverse this resistance is an urgent issue.

Mitogen-activated protein kinase kinase (MEK) plays an important role in many cellular processes, including proliferation, differentiation and development, primarily via activating the ERK cascade [12]. Despite being primarily localized in the cytoplasm due to its N-terminal nuclear export signal (NES) [13], MEK actually undergoes rapid shuttling in and out of the nucleus, which is enhanced by mitogenic stimulation [14]. However, the impacts and the regulatory mechanisms of this translocation still remain largely unknown.

Beta-transducin repeats-containing proteins (β-TrCP) serve as substrate recognition subunits for β-TrCP-SCF E3 ubiquitin ligases [15]. YAP, the main downstream effector of Hippo pathway, is involved in tissue regeneration, organ size control, stem cell self-renewal and tumourigenesis [16]. The Hippo kinase cascades MST/LATS phosphorylate YAP on multiple sites, resulting in its inactivation through cytoplasmic sequestration by 14-3-3 binding [17] and degradation by β-TrCP binding [18].

In this study, we identified that MEK nuclear localization could sequester β-TrCP from cytoplasmic inactive YAP, then stabilizing YAP. Tissue microarray analysis in colon tumours showed that aberrant MEK nuclear localization was associated with YAP expression and tumour malignancy. Interestingly, we found that the allosteric MEK inhibitor trametinib also inhibited MEK nuclear translocation, and then promoted YAP degradation. Mutant KRAS could restrain MEK within the cytoplasm via a scaffold protein IQ motif containing GTPase-activating protein 1 (IQGAP1), resulting in YAP downregulation. As a consequence, combination of KRAS inhibition and trametinib effectively suppressed the viability of KRAS mutant colon cancer cells in vitro and in vivo.

To investigate the underlying mechanisms of mutant KRAS target drugs resistance and the regulation of YAP by MEK translocation, most experiments were performed in the KRAS mutant colon cell lines SW1116 and SW480 and in the KRAS mutant breast cancer cell line MDA-MB-231 with a homozygous mutation in NF2 (also known as merlin), which, thus was in a Hippo/LATS-off condition at baseline [19].

## Results

### MEK nuclear translocation promotes β-TrCP nuclear localization

**Fig. 1:**
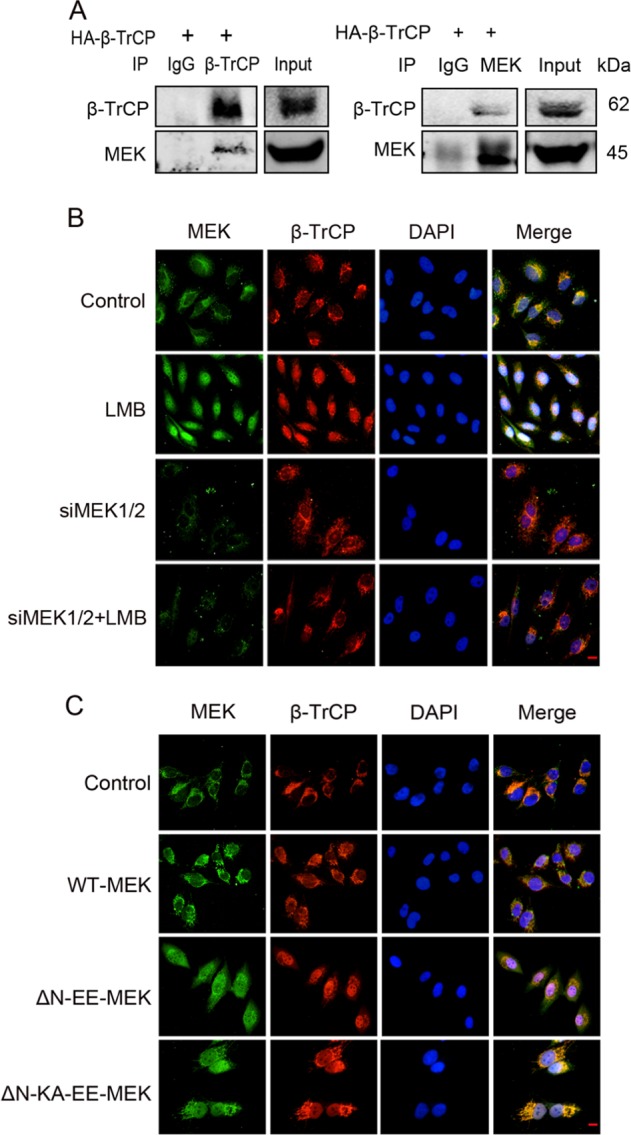
MEK nuclear translocation promotes β-TrCP localization in the nucleus. MEK localization was visualized by immunofluorescence staining with anti-MEK1/2 antibody (green). β-TrCP was shown by anti-β-TrCP staining (red). DNA was stained with DAPI (blue). Scale bar: 25 μm. **a** SW1116 cells were transfected with HA-β-TrCP. Anti-TrCP or anti-MEK1/2 antibody was used for IP. Blots were probed with anti-β-TrCP and anti-MEK1/2. **b** MDA-MB-231 cells were transfected with siMEK1/2 or negative control. Seventy-two hours after transfection, the cells were treated with LMB (10 ng/ml, 2 h) or left untreated. **c** MDA-MB-231 cells were transfected with control vetor, WT-MEK, ΔN-EE-MEK, ΔN-KA-EE-MEK. Seventy-two hours after transfection, the cells were treated with fresh medium containing 10%FCS for 6 h before IF

Using co-immunoprecipitation (co-IP), we found an unexpected interaction between MEK and β-TrCP in SW1116 cells (Fig. 1a). IF assays showed that β-TrCP co-localized with MEK in the cytoplasm of MDA-MB-231 and SW1116 cells (Fig. 1b and S1A). After LMB treatment, an inhibitor of NES-dependent nuclear export [20], MEK and β-TrCP exhibited primarily nuclear distribution (Fig. 1b and S1A). As no nuclear export signal for β-TrCP has been described, it was important to determine whether β-TrCP mislocalization was induced by MEK. After knocking down MEK1/2, β-TrCP mainly localized to the cytoplasm upon LMB treatment (Fig. 1b and S1A).

To directly prove that MEK localization could impair β-TrCP distribution, ΔN-S218ES222E-MEK (ΔN-EE-MEK, a NES-disrupted constitutively active MEK that directly localized to the nucleus) was transfected into SW1116 and MDA-MB-231 cells, subsequently β-TrCP became primarily nuclear localization (Fig. 1c and S1B). A small portion of β-TrCP remained localized to the cytoplasm, possibly because of the interaction with endogenous wild-type MEK. Since ΔN-EE-MEK could activate ERK, a NES-disrupted catalytically inactive MEK, ΔN-K97A-EE-MEK was transfected into cells. Most of ΔN-KA-EE-MEKs shifted to the nucleus when cells were cultured in complete medium and localized to the cytoplasm in serum-free medium [14]. After ΔN-KA-EE-MEK transfection in complete medium, a portion of β-TrCP also accumulated in the nucleus (Fig. 1c and S1B). These data provide evidence that MEK nuclear translocation could promote β-TrCP localization in the nucleus, possibly by acting as a chaperone, escorting β-TrCP in and out of the nucleus.

### MEK translocation regulates YAP stability by altering β-TrCP subcellular localization

YAP is phosphorylated by the Hippo/LATS kinase cascade, at least in the Ser127 and Ser397 sites, resulting in YAP cytoplasmic retention and subsequent binding to β-TrCP followed by degradation [18].

**Fig. 2:**
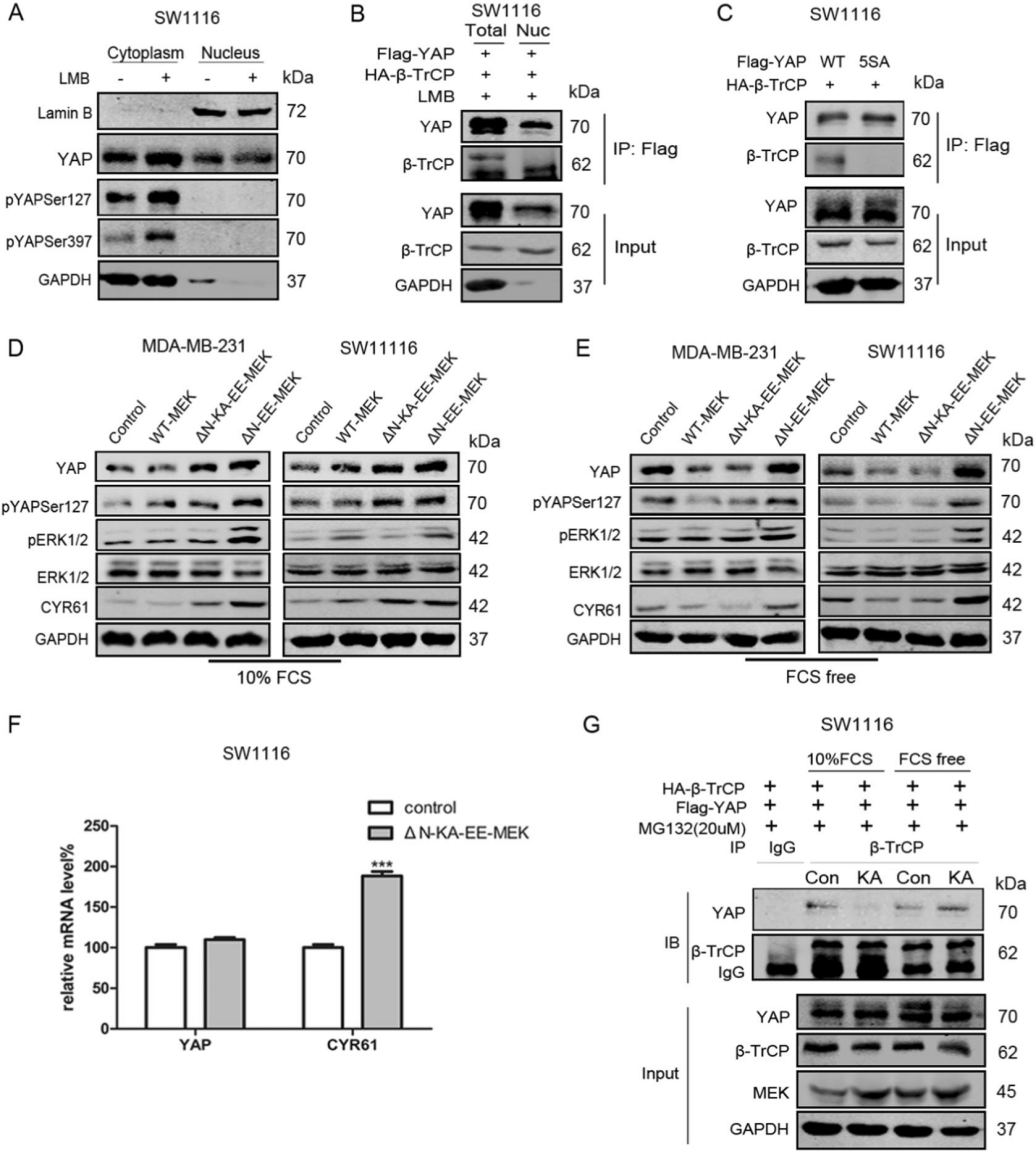
MEK translocation regulates YAP stability via modifying β-TrCP subcellular localization. **a** Extracting the cytoplasmic and nuclear protein, then WB for YAP, pYAPSer127 and pYAPSer397 in SW1116 cells treated with DMSO or LMB (10 ng/ml, 4 h). **b** Separating the nucleus by Nuclear Extraction Kit. Cell lysis buffer for IP was used to lyse nucleus and total cells; SW1116 cells were transfected with HA-β-TrCP and FLAG-YAP. Anti-FLAG antibody was used for IP. Blots were probed with anti-TrCP and anti-YAP. **c** SW1116 cells were transfected with HA-β-TrCP and FLAG-YAP or FLAG-YAP5SA. Anti-FLAG antibody was used for IP. Blots were probed with anti-TrCP and anti-YAP. **d** Western blotting for YAP and CYR61 in MDA-MB-231 and SW1116 cells transfected with control vetor, WT-MEK, ΔN-EE-MEK, ΔN-KA-EE-MEK for 72 h. The cells were treated with fresh medium containing 10%FCS for 6 h before extracted protein. **e** Western blotting for YAP and CYR61 in MDA-MB-231 and SW1116 cells transfected with control vetor, WT-MEK, ΔN-EE-MEK, ΔN-KA-EE-MEK for 72 h. The cells were treated with FCS-free medium for 16 h before extracted protein. **f** Quantitative real-time RT–PCR to measure YAP and CYR61 mRNA level in SW1116 cells. The cells were transfected with control vetor or ΔN-KA-EE-MEK. Seventy-two hours after transfection, the cells were treated with fresh medium containing 10%FCS for 6 h. GAPDH was used as a control. ***p < 0.001 using Student’s *t* test (two-tailed). **g** SW1116 cells were transfected with FLAG-YAP and HA-β-TrCP with or without over-expressing ΔN-KA-EE-MEK. MG132 (20 μM, 10 h) was used to prevent YAP degradation. Anti-TrCP antibody was used for IP. Blots were probed with anti-TrCP and anti-YAP

We first assessed that pYAPSer127 and pYAPSer397 were localized in the cytoplasm of SW1116 cells by WB (Fig. 2a), even after LMB treatment [21]. YAP5SA, with five LATS phosphorylation sites mutated to alanines, primarily localized to the nucleus. With co-IP in SW1116 cells, no nuclear YAP-β-TrCP or YAP5SA-β-TrCP interaction was detected (Fig. 2b, c), indicating that YAP recognition by β-TrCP occurred in the cytoplasm. It became interesting to investigate whether MEK translocation could regulate YAP stability via altering β-TrCP localization. We found ΔN-EE-MEK dramatically increased YAP and its target gene CYR61 protein levels (Fig. 2d). Despite ΔN-KA-EE-MEK being unable to activate ERK, it still upregulated YAP expression (Fig. 2d). Since ΔN-KA-EE-MEK and WT-MEK primarily localized to the cytoplasm in resting cells [14], so cells were treated with serum-free medium after transfection with WT-MEK or ΔN-KA-EE-MEK to validate the effect of cytoplasmic MEK on YAP levels, and then YAP levels were decreased (Fig. 2e).

The changes of pYAPSer127 and YAP levels were consistent in trend (Fig. 2d and e). We also knocked down LATS1/2 in SW1116 cells in complete medium and found that ΔN-KA-EE-MEK transfection still upregulated YAP expression (Fig. S2A). Both of MDA-MB-231 and SW1116 cells exhibited abnormal YAP nuclear localization and showed no difference in YAP localization after transfecting with ΔN-KA-EE-MEK (Fig. S2B). These data, especially those obtained from the Hippo/LATS inactive cell line MDA-MB-231, indicated that regulation of YAP by MEK translocation is independent of Hippo/LATS.

QPCR assays revealed that the mRNA levels of YAP were unaffected by ΔN-KA-EE-MEK, while the mRNA levels of CYR61 were significantly increased (Fig. 2f). By co-IP, the interaction between YAP and β-TrCP was reduced after ΔN-KA-EE-MEK transfection in SW1116 cells cultured in complete medium (Fig. 2g). Similar results were also obtained from cells treated with LMB (Fig. S2C). Over-expression of β-TrCP could reverse ΔN-KA-EE-MEK induced YAP upregulation (Fig. S2D). However, after refreshing with serum-free medium, ΔN-KA-EE-MEK transfection instead increased the binding of β-TrCP to YAP (Fig. 2g).

Taken together, these data suggest that MEK nuclear translocation stabilizes YAP by sequestering β-TrCP from inactive YAP, while cytoplasmic MEK restrains β-TrCP in the cytoplasm and promotes YAP degradation.

### Trametinib downregulates YAP via inhibition of MEK nuclear localization

**Fig. 3:**
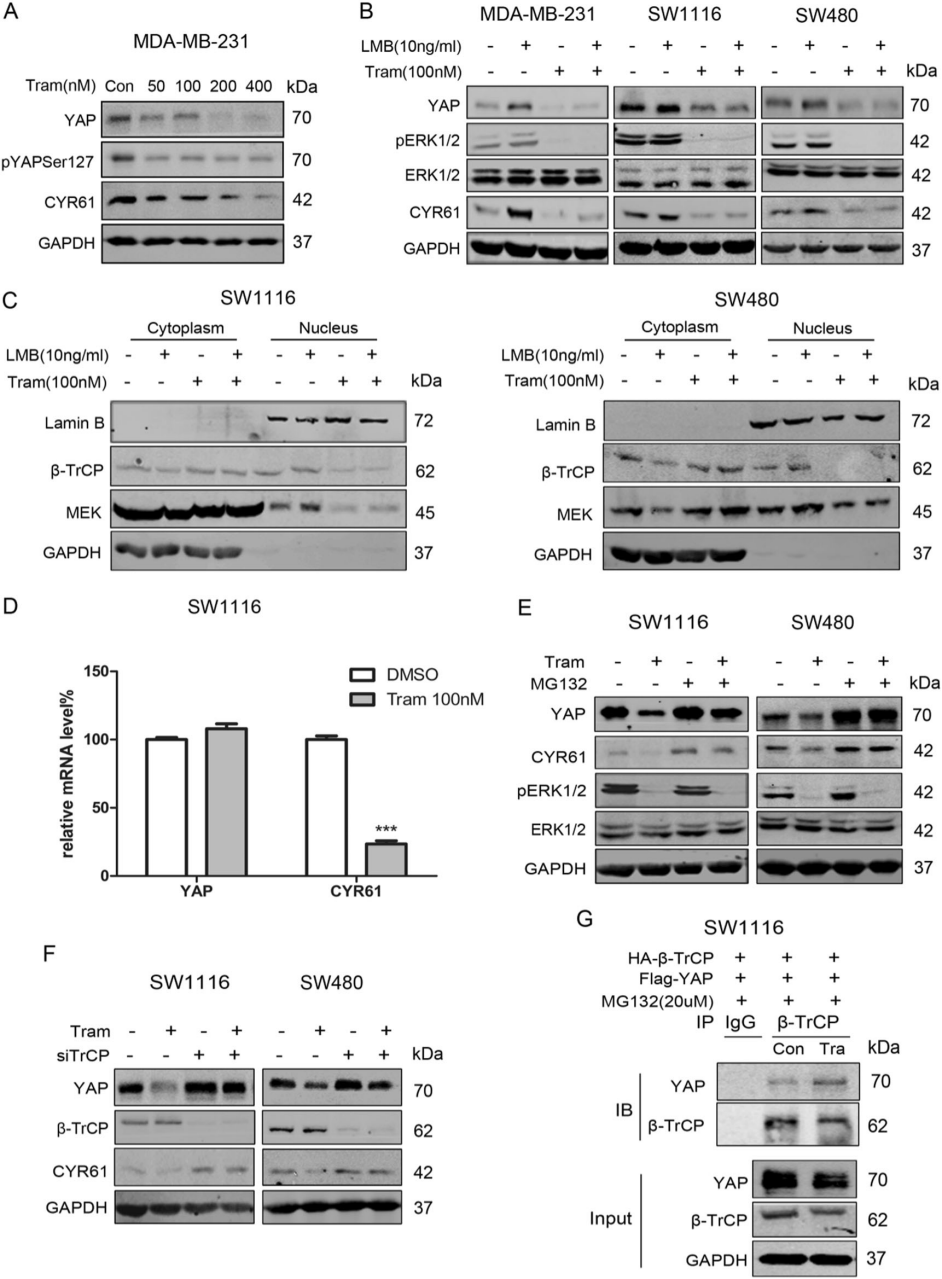
Trametinib downregulates YAP via inhibiting MEK nuclear localization. **a** Western blotting for YAP and CYR61 in MDA-MB-231 cells treated with increasing concentration of trametinib (50–400 nM). **b** WB for YAP and CYR61 using MDA-MB-231, SW480 and SW1116 cells treated with LMB (10 ng/ml, 4 h) or left untreated in the presence or absence of trametinib (100 nM, 24 h). **c** Extracting the cytoplasmic and nuclear protein, then WB for MEK and β-TrCP in SW1116 and SW480 cells treated with LMB (10 ng/ml, 4 h) or left untreated in the presence or absence of trametinib (100 nM, 24 h). **d** Quantitative real-time RT–PCR to measure YAP and CYR61 mRNA levels in SW1116 cells treated with trametinib (100 nM, 24 h) or DMSO. GAPDH was used as a control. ***p < 0.001 using Student’s *t* test (two-tailed). **e** WB for YAP and CYR61 using SW1116 and SW480 cells treated with trametinib (100 nM, 24 h) or DMSO supplemented with or without MG132 (20 μM) for 10 h. **f** WB for YAP, CYR61 and β-TrCP using SW1116 and SW480 cells treated with trametinib (100 nM, 24 h) or DMSO combined with or without siTrCP. **g** SW1116 cells were transfected with FLAG-YAP and HA-β-TrCP, then treated with trametinib (100 nM, 24 h) or left untreated. MG132 (20 μM, 10 h) was used to prevent YAP degradation. Anti-TrCP antibody was used for IP. Blots were probed with anti-TrCP and anti-YAP

Trametinib, a selective allosteric inhibitor of MEK kinase, has been clinically used to treat metastatic melanoma and non-small-cell lung cancer harbouring BRAF V600E mutations [22, 23]. Interestingly, trametinib could decrease YAP levels and inhibit LMB-induced YAP upregulation in MDA-MB-231, SW1116 and SW480 cells (Fig. 3a, b). Further study showed that trametinib reduced the expression of nuclear MEK/β-TrCP and inhibited the upregulation of nuclear MEK/β-TrCP induced by LMB in SW1116 and SW480 cells (Fig. 3c). Similar results were found by IF assays in MDA-MB-231 cells and SW480 cells (Fig. S3A, S3B).

QPCR assays in SW1116 cells showed that trametinib decreased YAP expression at the post-transcriptional level (Fig. 3d). These findings were confirmed by the observation that the effects of trametinib were reversed by MG132 or β-TrCP siRNA in SW1116 and SW480 cells (Fig. 3e, f). Moreover, co-IP results directly showed that trametinib increased the interaction between β-TrCP and YAP (Fig. 3g, S2C).

In conclusion, these data suggest that trametinib could increase cytoplasmic MEK/β-TrCP levels via inhibiting MEK nuclear translocation, promoting β-TrCP binding to YAP and subsequently YAP degradation.

### Mutant KRAS acts through IQGAP1 to restrain MEK in the cytoplasm

**Fig. 4:**
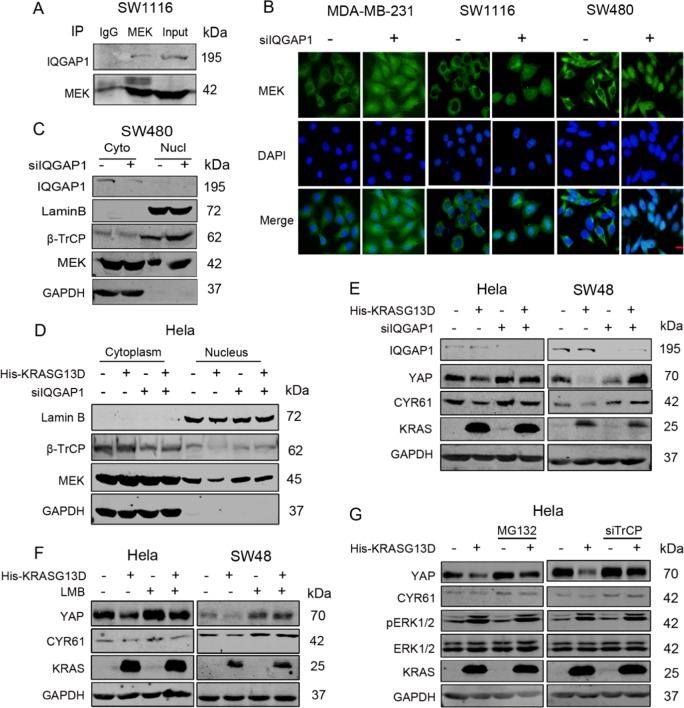
Mutant KRAS acts through IQGAP1 to restrain MEK in the cytoplasm. **a** Anti-MEK1/2 was used for IP in SW1116 cells. Blots were probed with anti-MEK1/2 and anti-IQGAP1. **b** MDA-MB-231, SW1116 and SW480 cells were transfected with siIQGAP1 or negative control. And 60 h after transfection, MEK localization was visualized by IF staining with anti-MEK1/2 antibody (green). DNA was stained with DAPI (blue). Scale bar: 25 μm. **c** Extracting the cytoplasmic and nuclear protein, then WB for MEK, β-TrCP and IQGAP1 in SW480 cells transfected with siIQGAP1 or negative control. **d** Extracting the cytoplasmic and nuclear protein, then WB for MEK and β-TrCP in Hela cells transfected with control or KRASG13D vector in the presence or absence of IQGAP1 silencing. **e** WB for YAP and CYR61 in Hela and SW48 cells transfected with control or KRASG13D vector in the presence or absence of IQGAP1 silencing. **f** WB for YAP and CYR61 in Hela and SW48 cells transfected with control or KRASG13D vector in the presence or absence of LMB (10 ng/ml, 4 h). **g** WB for YAP and CYR61 in Hela cells transfected with KRASG13D vector or control vector in the presence or absence of β-TrCP silencing (or MG132 20 μM, 10 h)

The scaffold protein IQGAP1 directly binds to MEK through its IQ region and assembles RAF, MEK and ERK to facilitate their sequential activation [24, 25]. The interaction between MEK and IQGAP1 was confirmed by co-IP in SW1116 cells (Fig. 4a). IF assays showed that IQGAP1 knockdown could induce obvious MEK nuclear accumulation in MDA-MB-231, SW480 and SW1116 cells (Fig. 4b), although MEK and ERK kinases activities were decresded (Fig. S4A). This result was further confirmed by WB, showing that nuclear MEK and β-TrCP expressions were increased after siIQGAP1 transfection in SW480 cells (Fig. 4c).

YAP levels were also upregulated in MDA-MB-231, SW1116 and SW480 cells after IQGAP1 deletion (Fig. S4A). Transfection with another sequence of IQGAP1 siRNA also increased YAP expression in SW1116 and SW480 cells (Fig. S4B). The changes of pYAPSer127 and YAP levels were consistent in trend, and YAP localization was also unaffected (Fig. S4A and S2B), indicating the regulation independent of LATS. Trametinib eliminated the increased YAP levels induced by IQGAP1 silencing (Fig. S4C), further verifying that IQGAP1 knockdown could promote MEK nuclear translocation.

IQGAP1 silencing was also performed in KRAS wild-type Colo320, SW48 and Hela cells to determine whether this regulation was a general mechanism. No aberrant accumulation of nuclear MEK or increased YAP levels were found in these cells by IF and WB (Fig. S4A, S4D, S4E). However, IQGAP1 silencing could increase YAP levels in Hela and SW48 cells tranfected with KRASG13D (Fig. S4F). Those results indicated that regulation of MEK nuclear translocation by IQGAP1 might depend on mutant KRAS or cell types.

After transfection with KRASG13D vector in Hela cells, the expression of nuclear MEK and β-TrCP were reduced, while silencing of IQGAP1 completely recovered the decreased nuclear MEK and β-TrCP levels (Fig. 4d), indicating that MEK cytoplasmic retention by mutant KRAS was mediated by IQGAP1. Similarly, YAP levels were downregulated by KRASG13D, which can be also abolished by siIQGAP1 or LMB in Hela and SW48 cells (Figs. 4e, f).

The qPCR results showed that YAP mRNA levels did not change significantly after KRASG13D transfection in Hela cells (Fig. S4G), indicating that YAP was regulated at the post-transcriptional level. This finding was also confirmed by the results that YAP downregulation induced by KRASG13D was reversed by MG132 or si-TrCP (Fig. 4g).

We here show for the first time that deletion of IQGAP1 dramatically induces MEK nuclear translocation in KRAS mutant cancer cells, and that mutant KRAS could restrain MEK/β-TrCP in the cytoplasm via IQGAP1, then downregulating YAP expression.

### Inhibition of mutant KRAS promotes MEK nuclear localization

**Fig. 5:**
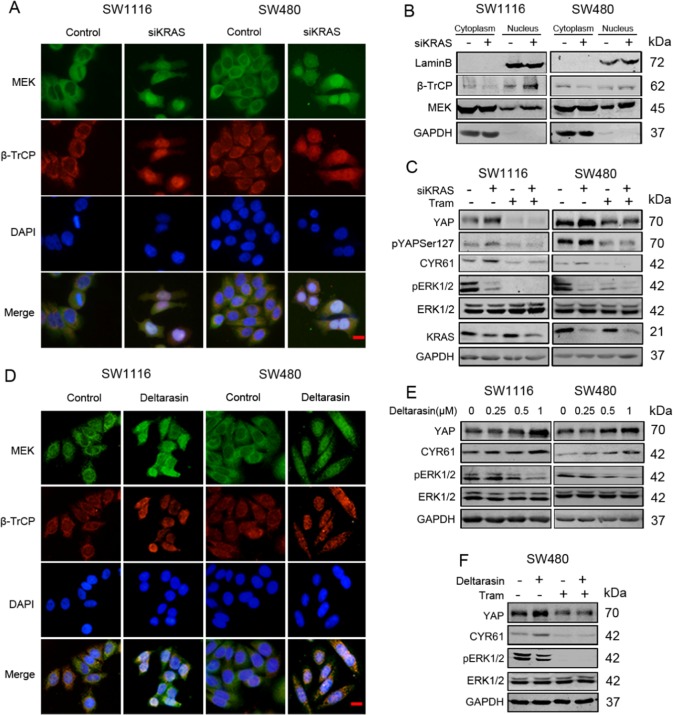
Mutant KRAS inhibition promotes MEK nuclear translocation. **a** SW1116 cells and SW480 cells were transfected with siKRAS or control. And 60 h after transfection, MEK and β-TrCP localization was visualized by IF staining with anti-MEK1/2 (green) and anti-β-TrCP (red). DNA was stained with DAPI (blue). Scale bar: 25 μm. **b** Extracting the cytoplasmic and nuclear protein, then WB for MEK and β-TrCP in SW1116 and SW480 cells transfected with siKRAS or negative control. **c** WB for YAP, CYR61 and KRAS in SW1116 and SW480 cells transfected with siKRAS or negative control in the presence or absence of trametinib (100 nM, 24 h). **d** SW1116 and SW480 cells were treated with deltarasin (1 μM, 12 h). MEK and β-TrCP localization was visualized by IF staining with anti-MEK1/2 (green) and anti-β-TrCP (red). DNA was stained with DAPI (blue). Scale bar: 25 μm. **e** WB for YAP and CYR61 in SW1116 and SW480 cells treated with the indicated concentrations of deltarasin (0.25–1 μM) or DMSO for 12 h. **f** WB for YAP and CYR61 in SW480 cells treated with deltarasin (1 μM, 12 h) in the presence or absence of trametinib (100 nM, 24 h)

After silencing KRAS in SW1116 and SW480 cells, MEK/β-TrCP nuclear localization were increased in SW1116 and SW480 cells by IF and WB (Fig. 5a, b). Despite the dramatically declined ERK kinases activities upon KRAS deletion, YAP levels were still increased, which was reversed by trametinib (Fig. 5c). Similar results were also observed in MDA-MB-231 cells by IF and WB (Fig. S5A, S5B). Another interference sequence targeting KRAS also increased YAP levels (Fig. S5C). The increased levels of pYAPSer127 and no obvious changes of YAP localization after KRAS deletion both indicated that the regulation was independent of LATS (Fig. S5C, S2B).

Deltarasin is a new small molecular inhibitor that suppresses oncogenic KRAS signalling by disrupting the binding of KRAS to its transporter PDEδ, preventing KRAS localizing to endomembranes [4]. Inhibition of mutant KRAS activity, cell growth, and tumour dissemination by deltarasin has been reported in pancreatic cancer, lung cancer and colon cancer [4, 26, 27]. By IF and WB assays, deltarasin had similar effects on MEK/β-TrCP nuclear localization and YAP levels (Fig. 5d, e). In addition, deltarasin-induced YAP upregulation was also prevented by trametinib treatment in SW480 cells (Fig. 5f).

We also performed KRAS silencing or deltarasin treatment in Hela cells, while no obvious MEK nuclear localization or increased YAP levels were detected (Fig. S5A, S5B, S5D), indicating that the regulation might depend on mutant KRAS or cell types. Taken together, these experiments provided evidence that mutant KRAS inhibition could promote MEK/β-TrCP nuclear localization to upregulate YAP levels.

### Aberrant nuclear localization of MEK and high expression of YAP in colon cancers

**Fig. 6:**
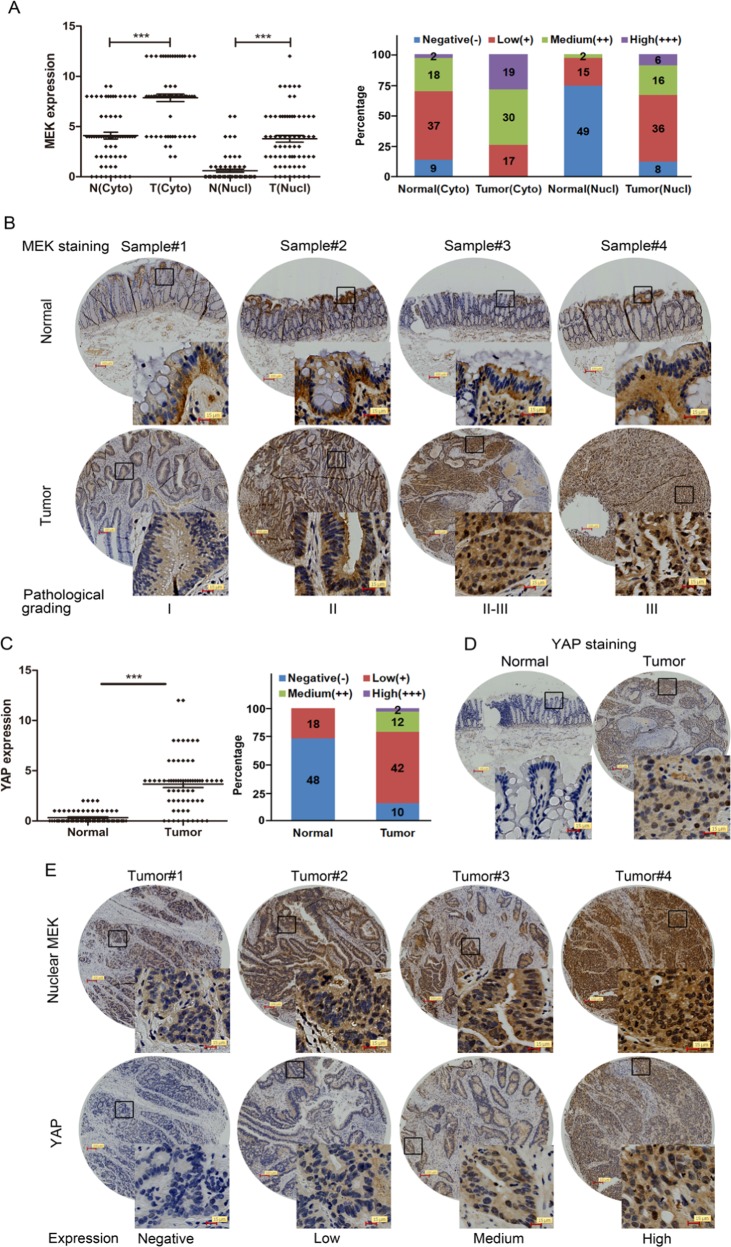
Aberrant nuclear localization of MEK and high expression of YAP in colon cancers. **a** TMA analysis of cytoplasmic and nuclear MEK expression in clinical samples of normal and colonl cancer tissues. IHC scores were showed in the left graph. The method assigning IHC scores for each sample was described in ‘Materials and methods' section. The bar graph (right) indicates the percentage of samples, ***p < 0.001. **b** Representative pictures of MEK staining in clinical samples of normal and colon cancer tissues with different pathological grading. Scale bar: 100/15 μm. **c** TMA analysis of YAP expression in normal and colon cancer tissues. IHC scores were showed in the left graph. The method assigning IHC scores for each sample was described in ‘Materials and methods' section. The bar graph (right) indicates the percentage of samples, ***p < 0.001. **d** Representative pictures of normal and colon cancer tissues stained for YAP. Scale bar: 100/15 μm. **e** Representative pictures of MEK and YAP staining in colon cancer tissues with different expression of nuclear MEK and YAP. Scale bar: 100/15 μm

Cytoplasmic and nuclear MEK levels were analysed in a colon cancer tissue microarray (TMA) containing 66 matched pairs of carcinoma and adjacent tissue samples by immunohistochemistry. Most tumour and normal tissues (>86%) both exhibited positive staining (+/+ +/+ + +) of cytoplasmic MEK, in which 74% (49/66) of tumour tissues displayed high expressions (+ +/+ + +) compared with 30% (20/66) of normal tissues (Fig. 6a, b). However, 88% (58/66) of tumours displayed positive nuclear MEK staining compared with only 26% (17/66) of normal samples, while 33% (22/66) of tumours and only 3% (2/66) of normal samples exhibited high expression (Fig. 6a, b).

To assess the clinical relevance of nuclear MEK, we analysed its correlation with clinicopathological parameters. The statistical results showed that no significant association was found between nuclear/cytoplasmic MEK expression and patient age, gender, tumour-staging, lymph node status, or tumour localization (Table S1). However, aberrant MEK nuclear localization was significantly associated with poor pathological grading (Table S1 and Fig. 6b), while no correction between cytoplasmic MEK expression and pathological grading.

Eighty-five percent (56/66) of tumours exhibited aberrant positive staining of YAP, compared with only 27% (18/66) of normal tissues (Fig. 6c, d). Interestingly, YAP expressions had a significant correlation with nuclear MEK staining, compared to a low correlation with cytoplasmic MEK levels (Table S2 and Fig. 6e).

Collectively, the statistical data of colon cancer TMA showed that nuclear MEK expression is more specifically detected in tumours, and closely associated with YAP expression and tumour malignancy. All the observations suggested that aberrant nuclear localization of MEK may contribute to the progression of colon cancer.

### Targeting KRAS synergizes with trametinib in suppressing the viability of KRAS mutant colon cancer cells

**Fig. 7:**
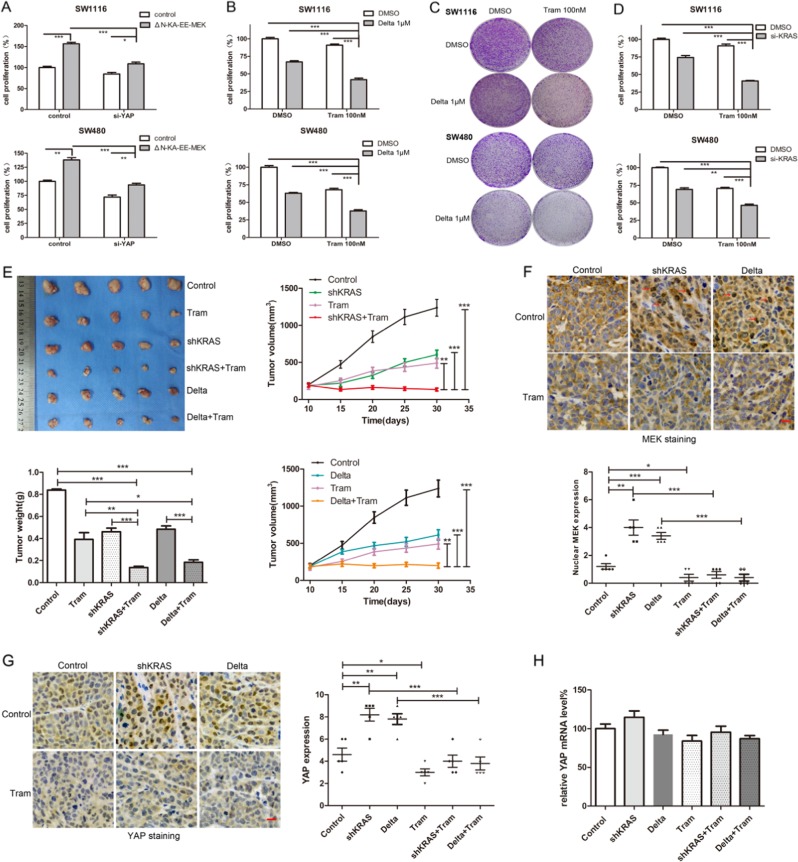
Targeting of KRAS synergizes with trametinib in suppressing the viability of KRAS mutant colon cancer cells in vitro and in vivo. **a** Cell proliferation assays using the Cell Counting Kit-8 in SW1116 and SW480 cells transfected with ΔN-KA-EE-MEK or control vector, in the presence or absence of YAP silencing at day 3. **b** Cell proliferation assays at day 3 of SW1116 and SW480 cells cultured with deltarasin (1M) or DMSO in the presence or absence of 100 nM trametinib. The data presented as the mean ± SD, *p < 0.05, **p < 0.01, ***p < 0.001 using Student’s *t* test (two-tailed). **c** Clonogenic assays of SW480 and SW1116 cells cultured with DMSO or 1 µM deltarasin (or/and 100 nM trametinib) at day 7. **d** Cell proliferation assays in SW1116 and SW480 cells transfected with siKRAS or negative control, and then cultured with 100 nM trametinib or DMSO for 3 days. The data presented as the mean ± SD, *p < 0.05, **p < 0.01, ***p < 0.001 using Student’s *t* test (two-tailed). **e** Tumour formation assays in the nude mice subcutaneously injected with SW480^Teto-shKRAS^ (group shKRAS and shKRAS + tram) and SW480^Teto-shControl^. The mice were treated with DMSO (group Control and shKRAS), deltarasin (15 mg/kg), trametinib (3 mg/kg) in the vehicle (20% PEG300, 5% Tween 80 and normal saline) according to groups via intraperitoneal injection daily. All groups were fed with doxycycline water (2 g/L). The bar graph indicates the tumour weight of each group (*n* = 5). The tumour sizes were measured every 5 days. The line charts show the tumour volume of each group (*n* = 5). The data presented as the mean ± SD, *p < 0.05, **p < 0.01, ***p < 0.001 using Student’s *t* test (two-tailed). **f** Representative pictures of MEK staining in xenograft tumour tissues. The graph indicates the IHC scores of nuclear MEK expression, *p < 0.05, **p < 0.01, ***p < 0.001 using Student’s *t* test (two-tailed). **g** Representative pictures of YAP staining in xenograft tumour tissues. The graph indicates the IHC scores of nuclear YAP expression, *p < 0.05, **p < 0.01, ***p < 0.001 using Student’s *t* test (two-tailed). **h** Quantitative real-time RT–PCR to measure YAP mRNA level in xenograft tumour tissues. GAPDH was used as a control, ***p *<* 0.001 using Student’s *t* test (two-tailed)

Since MEK nuclear localization had significant clinical relevance in colon cancer, we next investigated the effects of MEK nuclear translocation on cell proliferation. The results showed that ΔN-KA-EE-MEK strikingly promoted cell proliferation in SW1116 and SW480 cells, which was partially abolished by YAP knockdown (Fig. 7a). As mutant KRAS inhibition could promote MEK nuclear translocation, further CCK-8 and colony formation assays showed that inhibition of MEK nuclear localization by trametinib synergized with deltarasin or KRAS knockdown in inhibiting cell viability of SW1116 and SW480 cells (Fig. 7b, c, d). The HMG-CoA reductase inhibitor simvastatin has long been known to inhibit RAS activity by blocking RAS prenylation [28]. It also promoted MEK nuclear localization in SW1116, SW480 and MDA-MB-231 cells (Fig. S6A), although reports have noted that simvastatin could suppress YAP activity by inhibiting RhoA prenylation [29]. Simvastatin also synergized with trametinib in suppressing the viability of SW1116 and SW480 cells (Fig. S6B, S6C).

To futher validate the synergistic effect in vivo, we constructed a stable cell line SW480^Teto-shKRAS^, with a pTRIPZ lentiviral tetracycline-inducible (Tet-on) mir30shRNA (KRAS) vector that silenced KRAS in a doxycycline-dependent manner. The efficiency of doxycycline-induced KRAS knockdown was confirmed by WB (Fig. S6D). Tumour formation assays showed that trametinib synergized with KRAS knockdown or deltarasin in suppressing tumour growth (Fig. 7e). The efficiency of KRAS knockdown in tumours was confirmed by IHC (Fig. S6E). The percentage of Ki-67 positive cells was calculated by IHC and was lowest in the groups with combined treatment (Fig. S6F). Nuclear MEK and YAP staining were increased in tumours with KRAS knockdown or deltarasin treatment, which was also reversed by trametinib (Fig. 7f, g). YAP mRNA levels had no significant difference between groups (Fig. 7h). The body weights of mice showed no statistical difference between groups (Fig. S7).

## Discussion

### Regulation of MEK nuclear localization

Previous studies have suggested that MEK nuclear translocation might be dependent on the phosphorylation of its activation loop serines [14, 20]. The fact that ΔN-KA-EE-MEK but not ΔN-EE-MEK requires serum stimulation for the nuclear localization indicates that its nuclear localization may depend on endogenous MEK/ERK cascade. However, the nuclear translocation of MEK triggered by mutant KRAS/IQGAP1 inhibition indicated that the MEK/ERK kinase activities were dispensable. Some alternative mechanisms that promote MEK nuclear translocation might exist. Phosphorylation of a TPT motif was reported to directly mediate MEK nuclear translocation (similar mechanisms also mediate ERK2 nuclear translocation), although the specific kinases phosphorylating these sites were still unclear [30]. Recent studies showed that MEK has both allosteric and catalytic functions, and its activity could be triggered by the homodimerization and then intradimer transphosphorylation [31, 32]. Since ERK2 homodimerization is essential for its nuclear translocation [33], we speculate that the dimerization and intradimer transphosphorylation of MEK may also contribute to its nuclear localization. Such speculation may explain why trametinib could inhibit MEK nuclear localization, as Yuan et al. reported that trametinib could effectively impair the homodimers of MEK through an allosteric conformational change [31]. Further studies are needed to determine the upstream mechanisms that could directly promote MEK nuclear translocation.

Some scaffold proteins coordinating ERK/MAPK signalling could act as anchors to regulate MEK localization. It has been reported that Sef (similar expression to FGF) could restrain MEK in the cytoplasm [34]. Kinase suppressor of Ras (KSR) could interact with MEK and recruit MEK to the membrane [35]. The small GTPase RBJ acts as a nuclear anchor, mediating nuclear entrapment of MEK [36]. Our results describe that IQGAP1 could regulate MEK localization in KRAS mutant cancer cells. Some reports have noted that the components involved in ERK cascade transmission in KRAS mutant and wild cells are different [37, 38]. We suppose that mutant KRAS-driven ERK cascade tends to recruit more IQGAP1s to assemble pathway kinases [39, 40], resulting in MEK retention in the cytoplasm, which may explain why similar results were not obtained from some KRAS wild cells.

Ubiquitination and degradation of substrates such as YAP, TAZ and β-Catenin by β-TrCP were supposed to primarily occur in the cytoplasm [18, 41, 42]. In our research, MEK nuclear localization stabilizes YAP by sequestering β-TrCP in the nucleus, and inhibition of MEK nuclear localization by trametinib promotes YAP degradation. Two studies have reported similar results, showing that MEK inhibitors trametinib or PD98059 could downregulate YAP at the post-transcriptional level independent of LATS, but lacking further mechanistic study [43, 44]. Dysregulation of MEK nuclear localization is closely associated with YAP expression and tumour malignancy. Previous reports have shown that MEK nuclear localization could sustain nuclear ERK activity and promote tumour progression [34, 36]. These studies and our results all highlight the important role of MEK nuclear localization in tumourigenesis and tumour progression, which need to be further investigated systematically.

### The relationship between mutant KRAS and YAP

Activated KRAS are the only RAS type that could induce growth inhibition and apoptosis [45], indicating that mutant KRAS has both tumour-promoting and -suppressing functions. Kapoor et al. have reported that the gene coding for YAP is amplified when mutant KRAS slumbers in pancreatic cancer [8]. Another report showed that mutant KRAS could activate RASSF1A to downregulate YAP level in a lung cancer model [46]. Matallanas et al. observed similar results that mutant KRAS activates the pro-apoptotic MST2 kinase [47], which is supposed to inhibit YAP. However, Zhang et al. showed the opposite results that mutant KRAS induces post-transcriptional modification of YAP and augments its transcriptional activity in pancreatic cancer [48]. Gruber et al. found that KRASG12D upregulated YAP levels in acinar-to-ductal metaplasia lesions of a pancreatic tumour model [49]. Meanwhile, some other studies have noted that YAP levels are not affected by either mutant KRAS depletion or over-expression [9, 10]. These conflicting results indicate the complex regulation of YAP by mutant KRAS, which may differ in different tumours, different stages of tumourigenesis and even different mutant sites of KRAS.

### MEK nuclear translocation is a better drug target than YAP in combined treatment with KRAS inhibition

The capacity of YAP activation to bypass KRAS addiction has already been reported [8], we also confirmed it in our works (Fig. S8). But lacking of efficient small molecule inhibitors for YAP currently make it difficult to target. Thus, targeting MEK nuclear localization by clinical drug trametinib seems to be a better choice to overcome resistance to KRAS inhibition. Our results suggest that combination of KRAS inhibition and trametinib strikingly suppresses cell viability of KRAS mutant colon cancer cells. Similar results were obtained from HCT116 and MDA-MB-231 cells (Fig. S9A, S9B). However, no synergistic effect was detected in KRAS mutant HepG2 cell lines (Fig. S9C). Further study is needed to validate this combination in other tumours. Our results are consistent with a previous work that blocking the Raf/MEK/ERK pathway sensitizes cancer cells to statins [50]. In two recent works, the combination of trametinib and statins has also been identified as an effective therapy for tumours [51, 52]. However, for lack of efficacy in vivo with simvastatin [53, 54], the combination of simvastatin and trametinib in vivo was not performed.

**Fig. 8:**
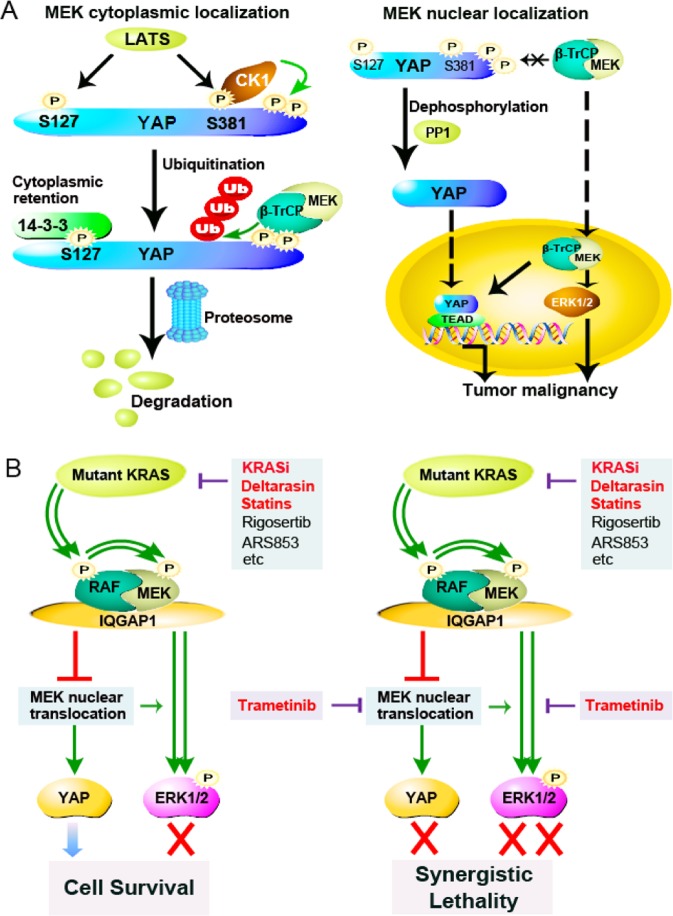
**a** The model of regulation of YAP by MEK localization. In resting cells, MEK localizes primarily in the cytoplasm, acting as a cytoplasmic anchor of β-TrCP, leading to YAP degradation. Upon stimulation, MEK translocates to the nucleus with β-TrCP, separating β-TrCP from cytoplasmic phosphorylated YAP. Then upon dephosphorylation by PP1, YAP is activated again and localizes to the nucleus. In addition, MEK localization in the nucleus could sustain nuclear ERK1/2 activity. Thus, the highly expressed YAP and nuclear phospho-ERK1/2 activated by MEK nuclear localization might together sustain tumour malignancy and promote tumour progression. **b** The model of combined therapy by targeting of KRAS and MEK nuclear translocation. In KRAS mutant colon cancer cells, highly active mutant KRAS triggers a complex of RAF/MEK/IQGAP1 resulting in MEK cytoplasmic retention. Targeting KRAS by inhibitors would result in MEK nuclear localization and YAP stability, helping tumours escape from mutant KRAS addiction. However, combined trametinib could downregulate YAP, and also enhance the inhibition of ERK1/2 activity, especially in the nucleus. Thus, combined targeting of KRAS and MEK nuclear translocation/YAP axis would effectively inhibit the survival of KRAS mutant colon cancers

In summary, our research suggests a novel function of MEK nuclear localization, wherein it sequesters β-TrCP in the nucleus to stabilize YAP and is closely associated with the malignancy of colon cancer (Fig. 8a). Meanwhile, we have identified a mutant KRAS/IQGAP1/MEK translocation/YAP axis and demonstrated that inhibition of KRAS promotes MEK nuclear translocation, subsequently stabilizing YAP (Fig. 8b). Therefore, combined targeting of KRAS and MEK nuclear translocation appears to be a promising therapeutic scenario in KRAS mutant colon cancers.

## Materials and methods

### Cell culture and reagents

MDA-MB-231 (KRASG13D), SW1116 (KRASG12A), SW480 (KRASG12V), Hela (KRASwt), SW48 (KRASwt) and Colo320 (KRASwt) cells were grown in Dulbecco’s modified Eagle medium with 10% foetal calf serum (FCS; all from Gibco/Invitrogen, USA). Trametinib and Deltarasin were from Selleckchem, Simvastatin was from Sigma, Leptomycin B was from Beyotime, China. MG132 and Doxycycline were from MedChemExpress. The following antibodies were used for WB, Co-IP, IF and IHC staining: Anti-YAP, anti-MEK1/2, anti-pERK1/2, anti-Lamin B, anti-IQGAP1 and anti-Ki-67 were all from Abcam. Anti-GAPDH and anti-HA were from Santa Cruz. Anti-LATS1 and anti-β-TrCP (WB/Co-IP) were from Cell Signaling Technology and anti-β-TrCP (IF) was from Sangon Biotech, China. Anti-pMEK1, anti-CYR61 and anti-Flag were from Sangon Biotech. Anti-KRAS was from Proteintech.

### Immunofluorescence staining

Cells were fixed with 4% paraformaldehyde for 15 min following permeabilization with 1% Triton X-100 for 15 min. After blocking in 3% bovine serum albumin (BSA) for 30 min, cells were incubated with primary antibody diluted in 1% BSA overnight at 4 °C. After washing with PBS, cells were incubated with Alexa Fluor 488- or 594-conjugated secondary antibodies (1:1000 dilution) for 1.5 h and then stained with 5 μg/ml DAPI for 5 min (Invitrogen) at room temperature. Immunofluorescence was detected using fluorescence microscope (Eclipse 80i, Nikon, Japan) at × 200 magnifications.

### Transfection

SiRNAs against MEK1/2, IQGAP1, KRAS, β-TrCP, YAP, LTAS1/2 and negative control were designed and synthesized by GenePharma. The sequences of siRNAs used in this study are provided in Fig. S11. The His-KRASG13D vector was constructed in our laboratory. The WT-MEK, ΔN-EE-MEK and ΔN-KA-EE-MEK vectors were gifts from Dr. Rony Seger (Weizmann Institute of Science, Rehovot, Israel). Flag-YAP, Flag-YAP5SA and HA-β-TrCP vectors were gifts from Dr. Bin Zhao (Life Sciences Institute, Zhejiang University, China). Lipofectamine 2000 (Invitrogen) was used for transfection according to the manufacturer’s instructions. The pTRIPZ lentiviral tetracycline-inducible (Tet-on) mir30shRNA (KRAS) vector was constructed by Obio Technology (Shanghai) and was transfected in cells according to protocol.

### RNA extraction, cDNA synthesis and quantitative real-time PCR

Total RNA was extracted using Trizol (Invitrogen) according to the manufacturer’s protocol and reverse transcribed into cDNA using the Reverse Transcriptase M-MLV (Takara, Dalian, China). Real-time PCR was performed using a SYBR Premix Ex Taq™ kit (Takara) on the iQ5 Real-Time PCR Detection System (Bio-Rad, Hercules, CA, USA). PCR primers used in this study are provided in Fig. S11. Gene expression levels for genes of interest were normalized to GAPDH and calculated as ΔC_T_ values (ΔC_T_ = C_T_ gene of interest – C_T_ GAPDH). Log2 fold changes in expression between treatment group samples and control group samples were calculated using the formula: log2 fold change = –ΔΔC_T_ = –[ΔC_T_ treatment group sample – ΔC_T_ control group sample].

### Immunoprecipitation assays and western blot analysis

Cells were lysed with IP lysis buffer (Beyotime). The whole-cell lysates were incubated with antibodies against Flag or β-TrCP overnight at 4 ℃, and then precipitated with the antibody protein complex using protein A/G beads (Selleck). The immunoprecipitates were washed five times, and then subjected to western blotting analysis. For western blot analysis, cells were lysed on ice using RIPA buffer (Beyotime) supplemented with protease and phosphatase inhibitors (Roche).

### Cell proliferation assays and colony formation assays

For cell proliferation assays, cells were seeded in 96-well plates 24 h. After transfection or treated with the indicated drugs, relative cell growth was measured using the Cell Counting Kit-8 (Dojingdo, Kumamoto, Japan). For colony formation assays, cells were seeded into 35 mm dish and cultured in the Dulbecco’s modified Eagle medium with 10% foetal calf serum overnight. Cells were then treated with drug as indicated in complete media for 7 days. Growth media with or without drug was replaced every 2 days. Remaining cells were fixed with methanol (1%) and formaldehyde (1%), stained with 0.5% crystal violet, and photographed using a digital scanner.

### In vivo xenograft experiments

Female BALB/c nude mice, 5–6 weeks old, were obtained from BeiJing HFK Bioscience. Mice were subcutaneously injected with SW480^Teto-shKRAS^ and SW480^Teto-shControl^ (1.0 × 10^7^ cells). Ten days after implantation, mice with tumour size of ~180 mm^3^ were subsequently assigned to six groups (*n* = 5), two groups with SW480^Teto-shKRAS^ (named shKRAS and shKRAS + tram) and four groups with SW480^Teto-shControl^ (named control, delta, tram and delta + tram). Mice were treated with DMSO (Group controls and shKRAS), deltarasin (15 mg/kg), or trametinib (3 mg/kg) in the vehicle (20% PEG300, 5% Tween 80 and normal saline) according to groups via intraperitoneal injection daily. All groups were fed doxycycline-containing water (2 g/L). Tumour diameters were serially measured with a digital calliper (Proinsa, Vitoria, Spain) every 5 days, and tumour volumes were calculated using the following formula: *V* = (L*W^2^)/2, where *L* and *W* represent length and width. All nude mice experiments were performed in accordance with the institute guidelines and were approved by the animal ethics committee of the China Institute of Science.

### Colon cancer tissue microarray

The human colon cancer tissue microarrays were prepared by Shanghai Outdo Biotech, China. All the patients signed informed consent forms. This study was approved by the Ethics Committee of Taizhou Hospital of Zhejiang Province.

### Immunohistochemistry

Immunohistochemistry (IHC) was performed on all human colon cancer samples and xenograft tumour tissues using biotin-streptavidin HRP detection systems. Each sample was assigned an IHC score based on the multiplied result of percentage positivity and staining intensity, and total scores ranged from 0 to 12. The percentage of positive cells was scored as 0 (<1%), 1 (1–25%), 2 (26–50%), 3 (51–75%), or 4 (>75%). Staining intensity was scored as 0 (no staining), 1 (weak staining), 2 (moderate staining), or 3 (strong staining). A total score of 0 indicated negative expression (−), 1–4 indicated low expression (+), 5–8 indicated medium expression (+ +) and 9–12 indicated high expression (+ + +).

### Statistical analysis

Student’s *t*-test (two-tailed) was used for comparisons between groups in cell proliferation assays and gene expression analysis by GraphPad Prism 5. Pearson’s *χ*^2^ test, Spearman’s rank correlation coefficient, and Yates’s chi-squared test were used for correlation analysis in SPSS. Statistical significance was set at *p < 0.05, **p < 0.01 and ***p < 0.001. No statistical methods were used to predetermine sample size. All experiments were performed using at least three biological replicates.

## Supplementary information

supplementary information
