# Supplementary material for: MEK nuclear localization promotes YAP stability via sequestering β-TrCP in KRAS mutant cancer cells
Source: Cell Death Differ. 2019 Mar 4;26(11):2400–15. doi: 10.1038/s41418-019-0309-6 (PMC6889282; doi:10.1038/s41418-019-0309-6)
Supplement: Supplementary file 1 — supplementary information [file 41418_2019_309_MOESM1_ESM.docx]

S1A


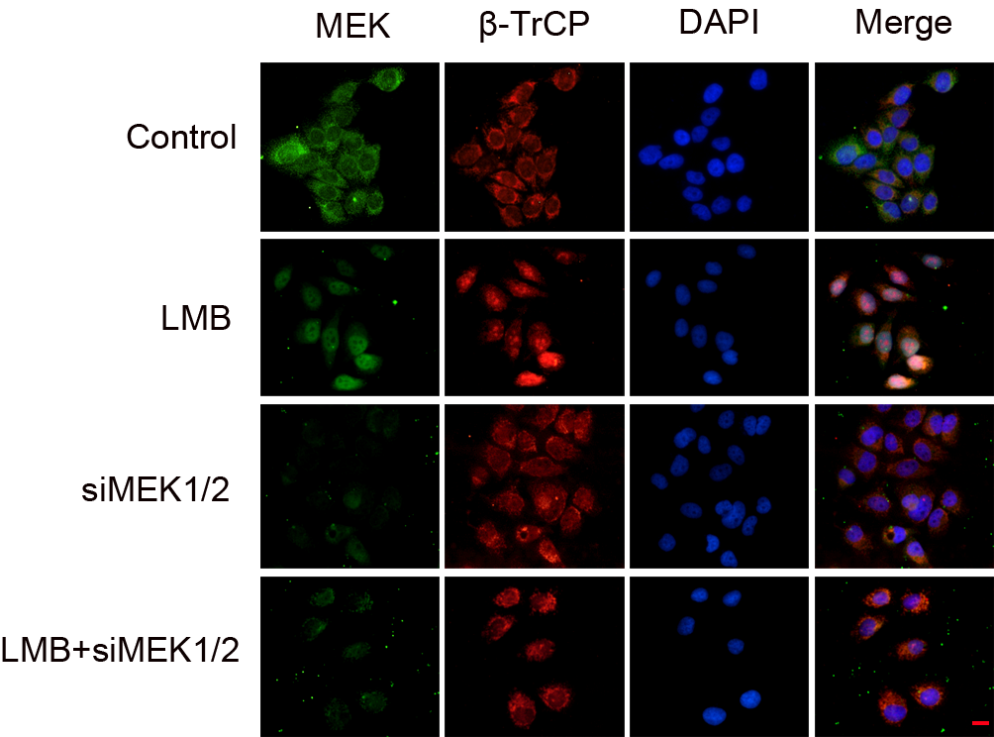


S1B


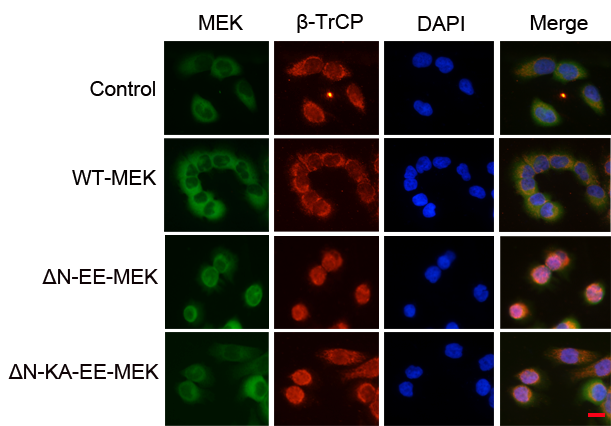


Figure S1: (A) SW1116 cells were transfected with siMEK1/2 or negative control. And 72h after transfection, the cells were were treated with LMB (10ng/ml, 2h) or left untreated. MEK localization was visualized by IF staining with anti-MEK1/2 antibody (green). β-TrCP was shown by anti-β-TrCP staining (red). DNA was stained with DAPI (blue). Scare bar: 25μm.

(B) SW1116 cells were transfected with control vetor、WT-MEK、ΔN-EE-MEK, ΔN-KA-EE-MEK. 72h after transfection, the cells were treated with fresh medium containing 10%FCS for 6h before IF. Scale bar: 25μm.

S2


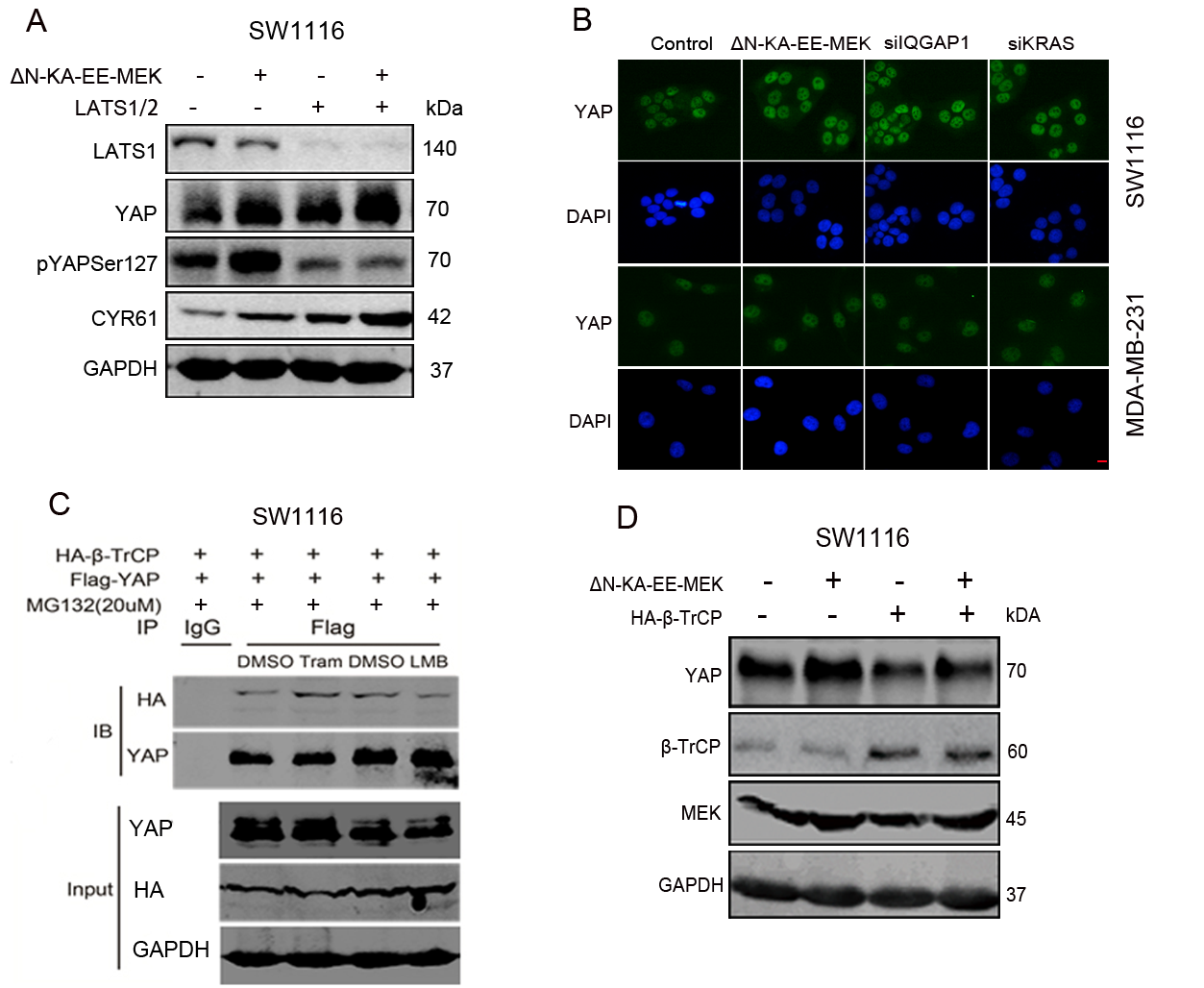


Figure S2: (A) Western blotting for YAP and pYAPSer127 in SW1116 cells transfected with ΔN-KA-EE-MEK or control vetor, in the presence or absence of siLATS1/2 silencing for 72h. The cells were treated with fresh medium containing 10%FCS for 6h before extracted protein.

(B) SW1116 and MDA-MB-231 cells were transfected with negative control, ΔN-KA-EE-MEK, siIQGAP1 or siKRAS. And 60h after transfection, YAP localization was visualized by IF staining with anti-YAP antibody (green). DNA was stained with DAPI (blue). Scare bar: 25μm.

(C) SW1116 cells were transfected with FLAG-YAP and HA-β-TrCP, then treated with trametinib (100nM, 24h) or LMB (10ng/ml, 4h) or left untreated. MG132 (20uM, 10h) was used to prevent YAP degradation. Anti-FLAG antibody was used for IP. Blots were probed with anti-HA and anti-YAP.

(D) Western blotting for YAP in SW1116 cells transfected with control vetor, ΔN-KA-EE-MEK, HA-β-TrCP or ΔN-KA-EE-MEK/HA-β-TrCP for 72h. The cells were treated with fresh medium containing 10%FCS for 6h before extracted protein.

S3A


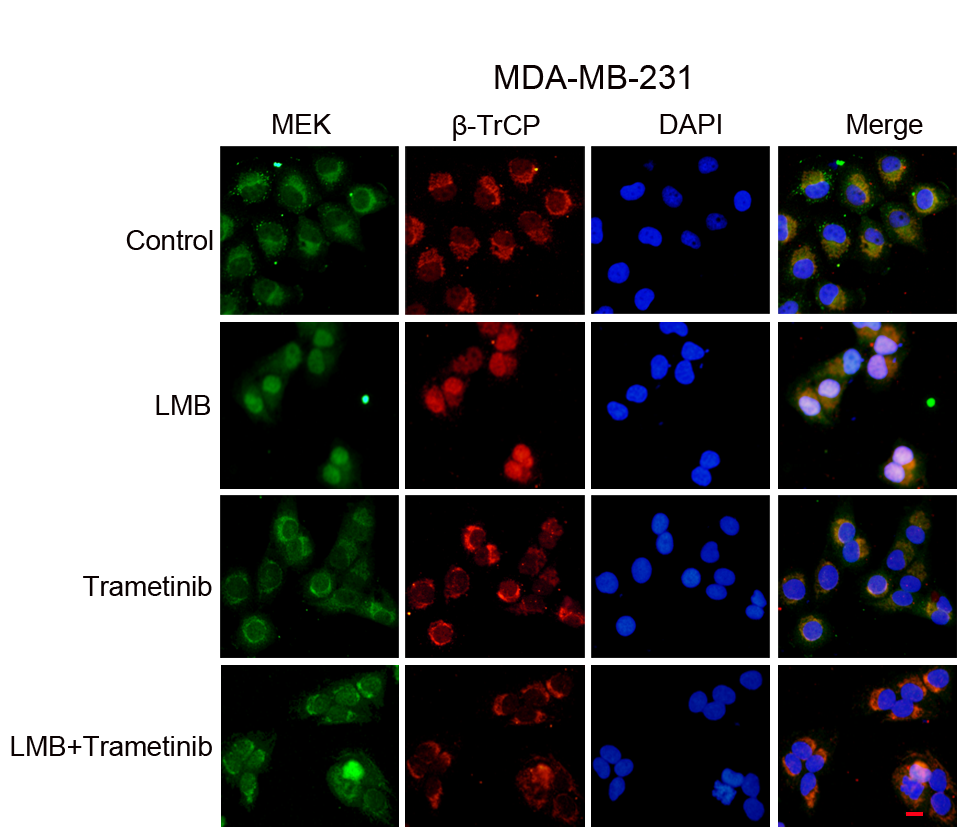


S3B


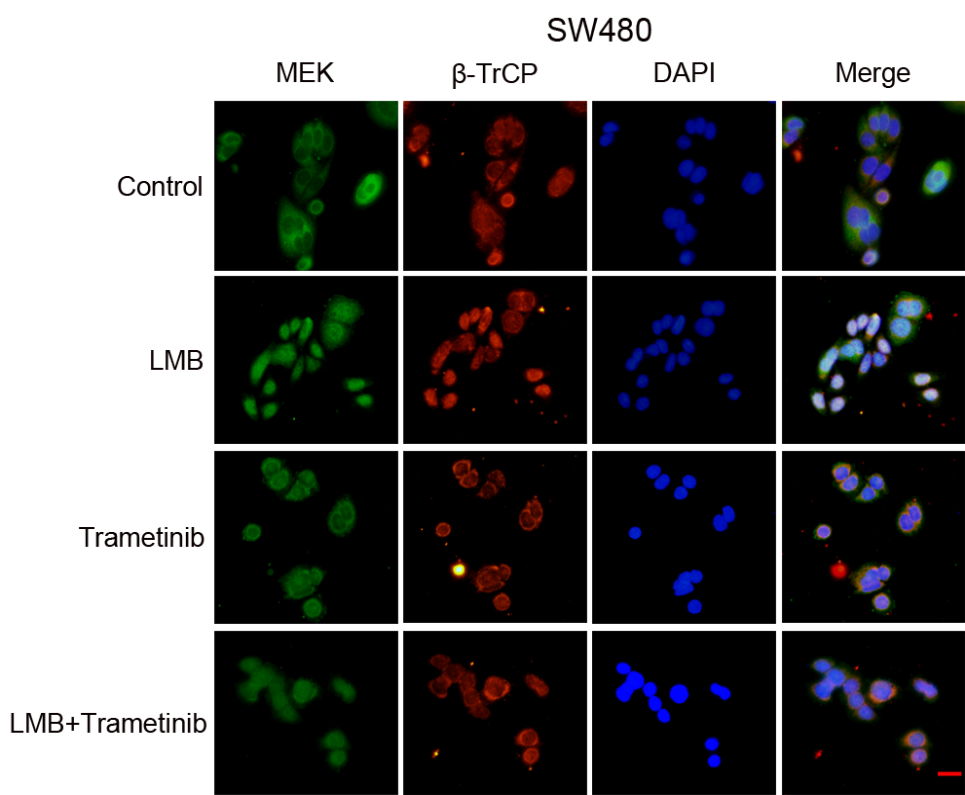


Figure S3: MDA-MB-231 and SW480 cells were treated with LMB (10ng/ml, 2h) or left untreated in the presence or absence of trametinib (100nM, 24h). MEK localization was visualized by IF staining with anti-MEK1/2 antibody (green). β-TrCP was shown by anti-β-TrCP staining (red). DNA was stained with DAPI (blue). Scale bar: 25μm.

S4


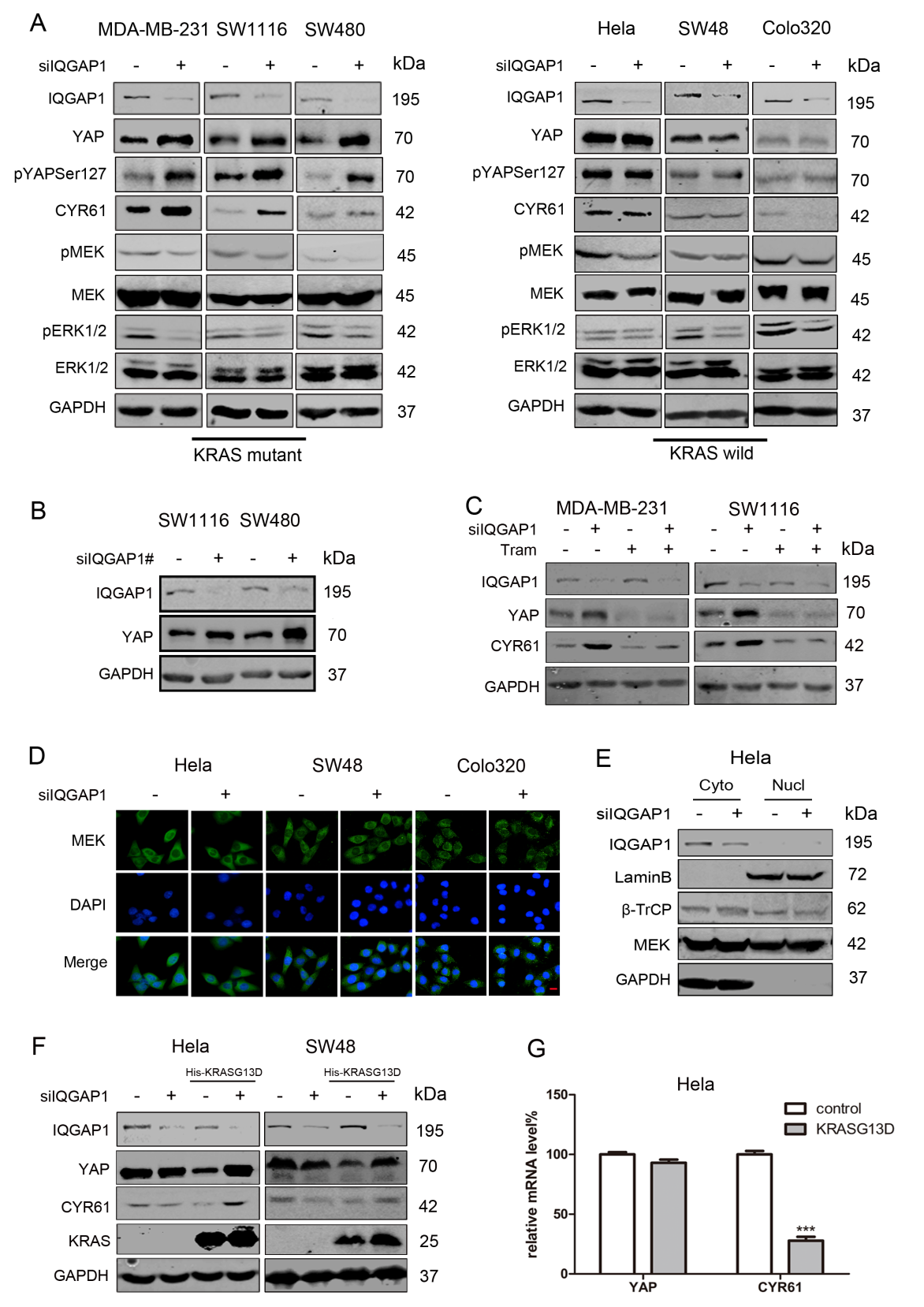


Figure S4: (A) WB for the indicated protein in KRAS mutant type SW1116, SW480, MDA-MB- 231 cells and KRAS wild type Hela, SW48, Colo320 cells with siIQGAP1 or negative control transfection for 60h.

(B) Western blotting for YAP and IQGAP1 in SW1116 and SW480 cells transfected with siIQGAP1# with another sequence.

(C) WB for YAP and CYR61 in MDA-MB-231 cells and SW1116 cells transfected with siIQGAP1 or negative control in the presence or absence of trametinib (100nM, 24h).

(D) Colo320, Hela and SW48 cells were transfected with siIQGAP1 or negative control. And 60h after transfection, MEK localization was visualized by IF staining with anti-MEK1/2 antibody (green).DNA was stained with DAPI (blue). Scale bar: 25μm.

(E) Extracting the cytoplasmic and nuclear protein, then WB for MEK, β-TrCP and IQGAP1 in Hela cells transfected with siIQGAP1 or negative control.

(F) WB for YAP and CYR61 in Hela and SW48 cells transfected with siIQGAP1 or negative control in the presence or absence of His-KRASG13D vector transfection.

(G) Quantitative real-time RT–PCR to measure YAP and CYR61 mRMA levels in Hela cells transfected with control or KRASG13D vector. GAPDH was used as a control. *** P < 0.001 using Student's t test (two-tailed).

S5


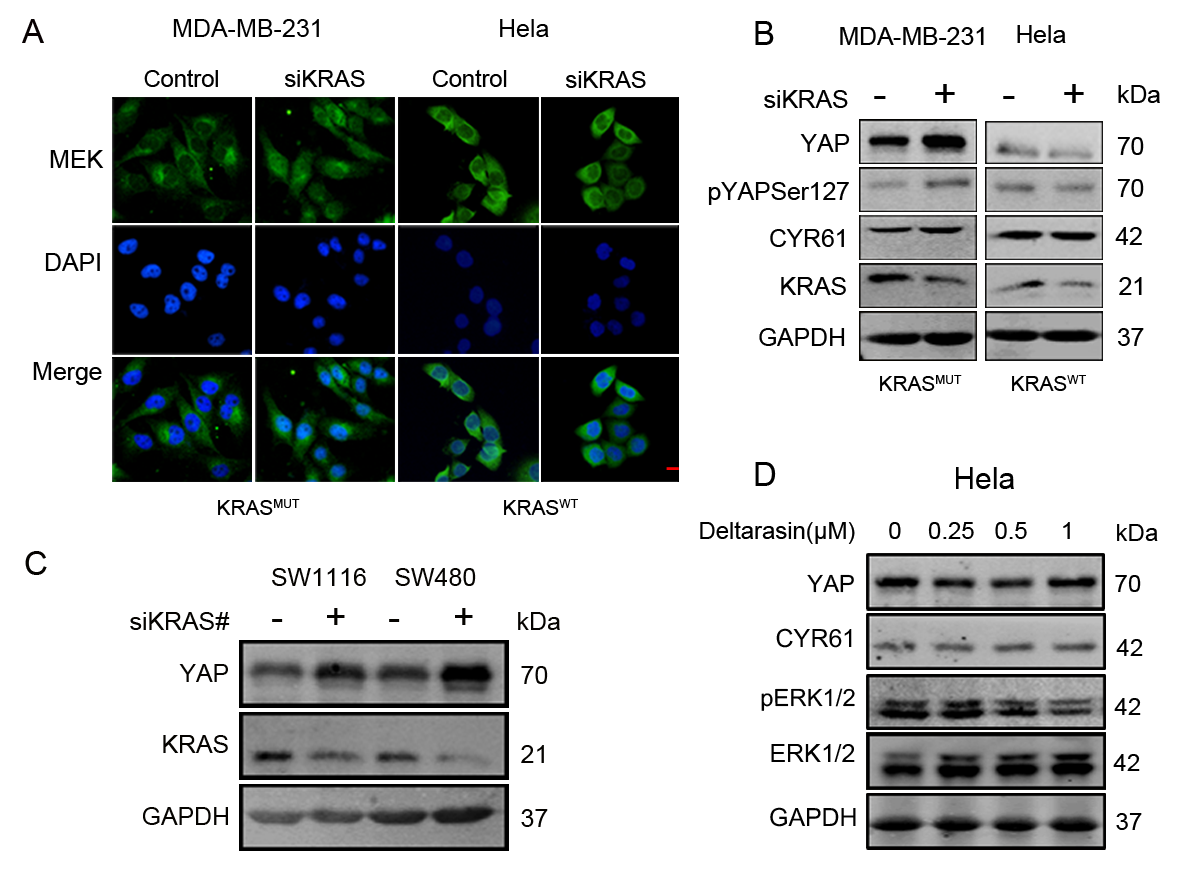


Figure S5: (A) KRAS mutant type MDA-MB-231 cells and KRAS wild type Hela cells were transfected with siKRAS or negative control. And 60h after transfection, MEK localization was visualized by IF staining with anti-MEK1/2 antibody (green). DNA was stained with DAPI (blue). Scale bar: 25μm.

(B) WB for YAP, pYAPSer127 and CYR61 in MDA-MB-231 and Hela cells.

(C) Western blotting for YAP and KRAS in SW1116 and SW480 cells transfected with siKRAS# with another sequence.

(D) WB for YAP and CYR61 in Hela cells treated with the indicated concentrations of deltarasin (0.25-1uM) or DMSO for 12h.

Table S1

Table S2

S6


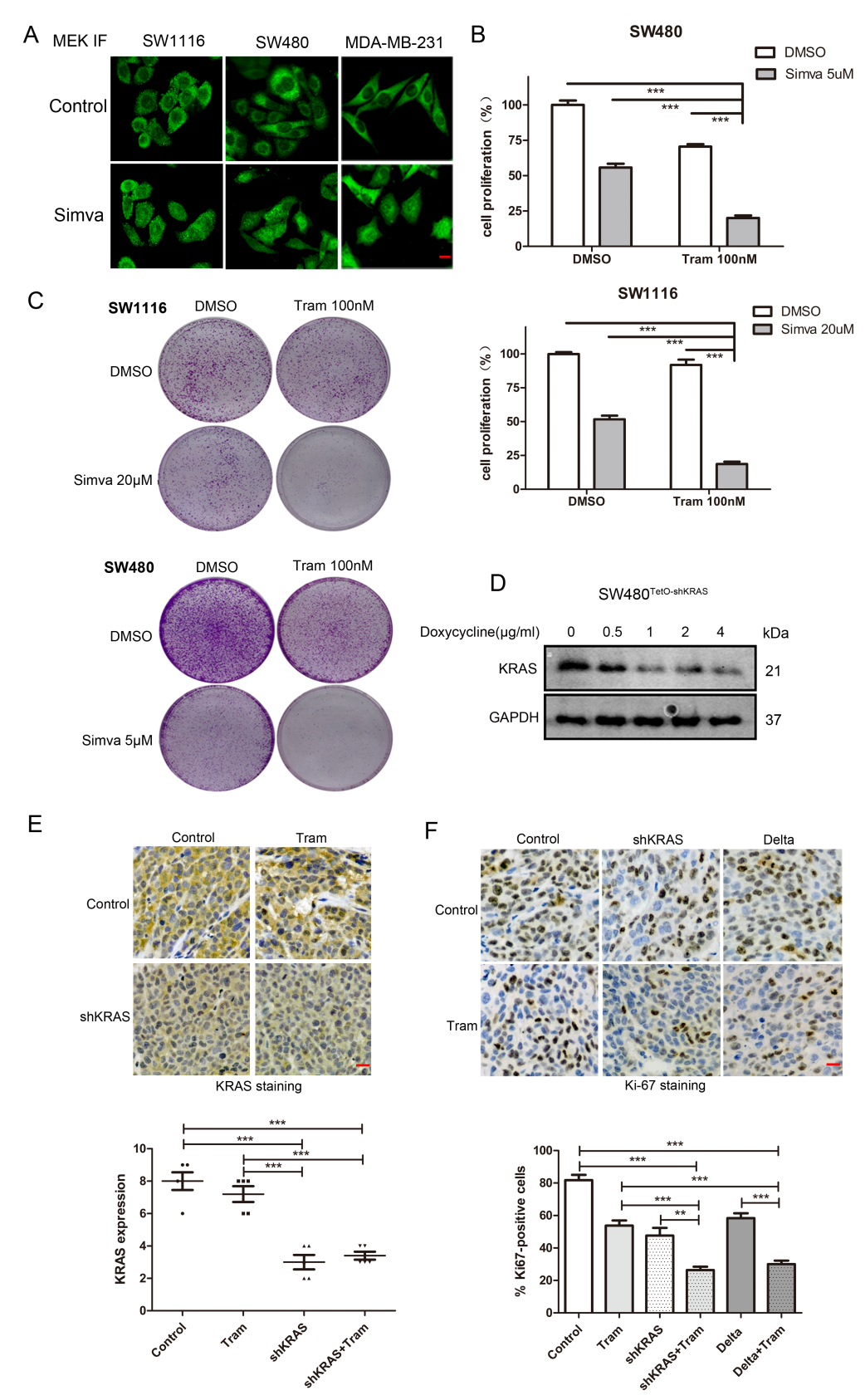


Figure S6: (A) SW1116, SW480 and MDA-MB-231 cells were treated with simvastatin (60uM, 6h). MEK localization was visualized by IF staining with anti-MEK1/2 antibody (green). Scale bar: 25μm.

(B) Cell proliferation assays at day 3 of SW1116 and SW480 cells cultured with simvastatin (20uM/5uM) or DMSO in the presence or absence of 100nM trametinib.

(C) Clonogenic assays of SW480 and SW1116 cells cultured with DMSO or 20uM/5uM simvastatin (or/and 100nM trametinib) at day 7.

(D) Western blotting for KRAS in SW480^Teto-shKRAS^ cells treated with increasing concentration of doxycycline (0.5-4 ug/ml).

(E) Representative pictures of KRAS staining in xenograft tumor tissues. The graph indicates the IHC scores of KRAS expression, **p* < 0.05, ***p* < 0.01, ****p* < 0.001 using Student's t test (two-tailed).

(F) Representative pictures of Ki-67 staining in xenograft tumor tissues. The bar graph indicates the percentage of Ki-67-positive cells, ***p* < 0.01, ****p* < 0.001 using Student's t test (two-tailed).

S7


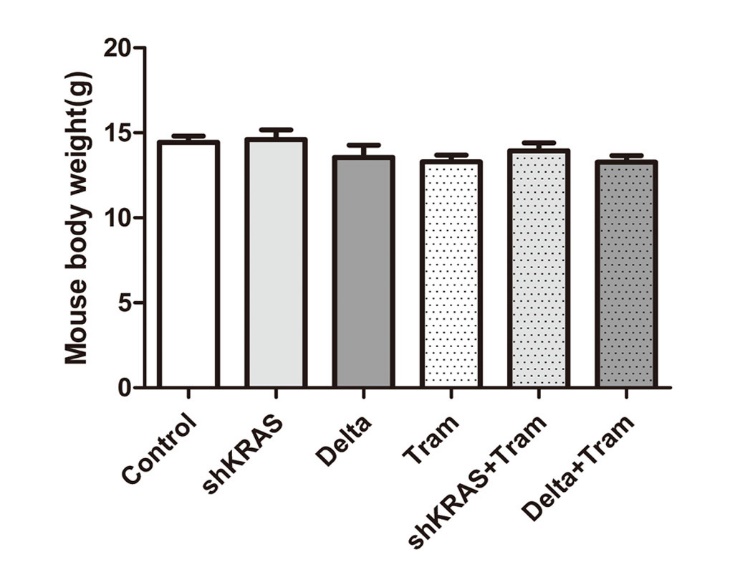


Figure S7: The bar graph indicates the mouse body weight of each group (n=5). The data presented as the mean±SD, showing no statistical significance between groups.

S8


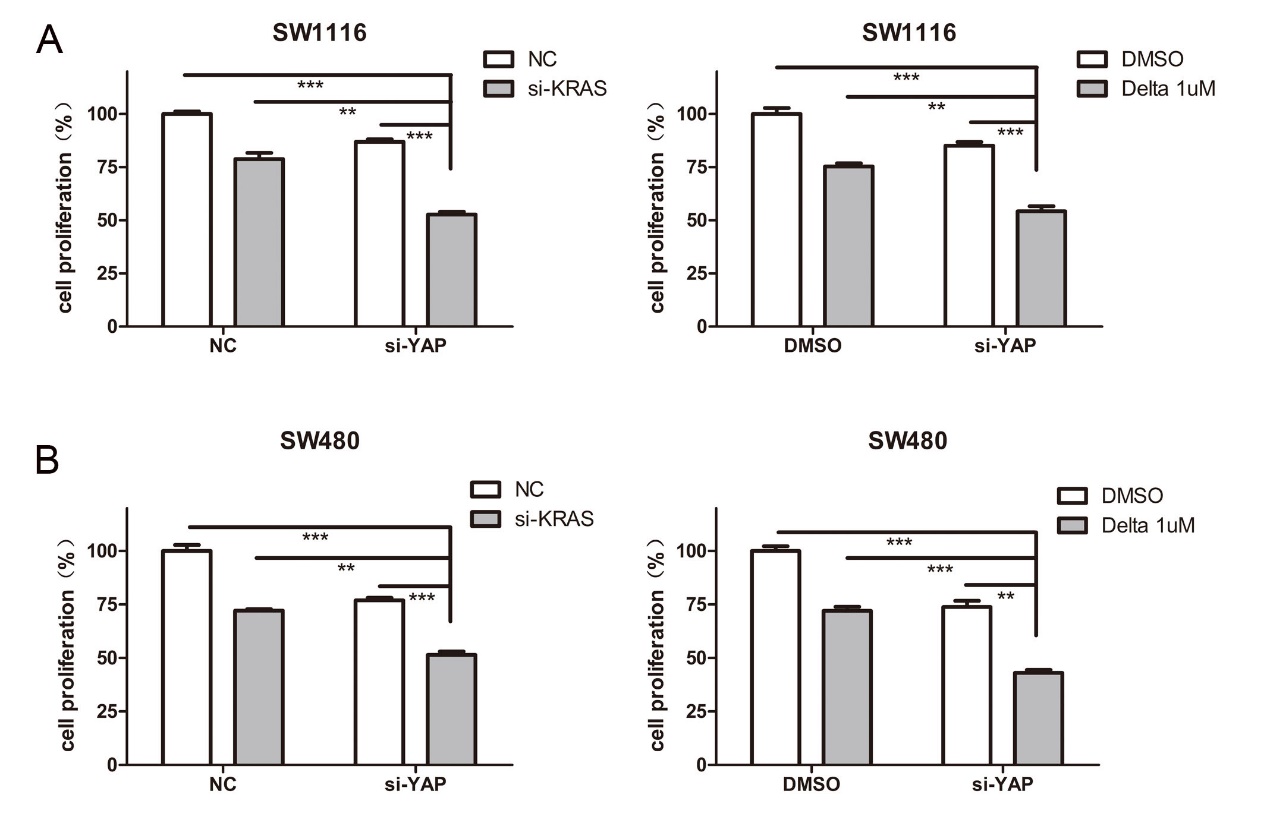


Figure S8: Cell proliferation assays of SW1116 and SW480 cells treated with si-KRAS, deltarasin or control in the presence or absence of YAP knockdown for 3 days. Data presented as mean ± SD. **P < 0.01, ***P < 0.001 using Student's t test.

S9


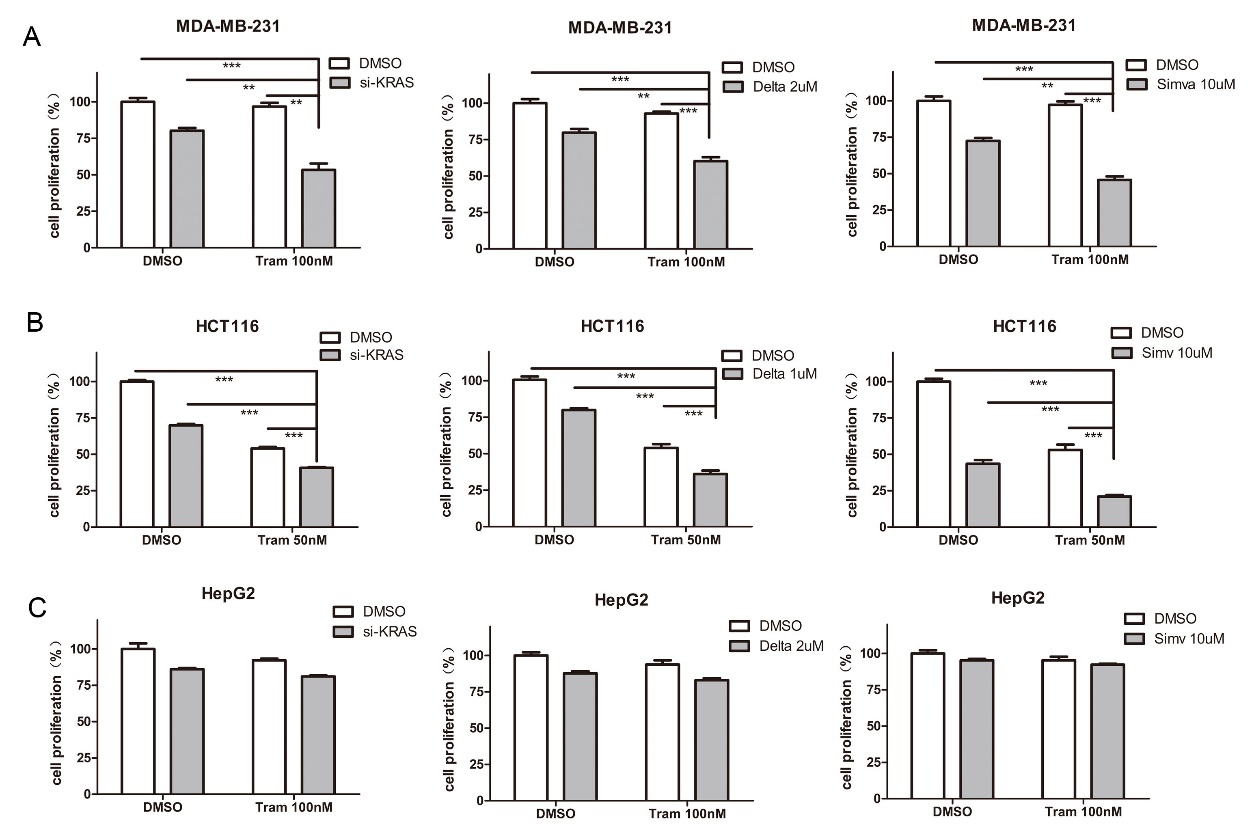


Figure S9: Cell proliferation assays of different cells treated with siKRAS, deltarasin, simvastatin or control in the presence or absence of trametinib for 3 days. Data presented as mean ± SD. **P < 0.01, ***P < 0.001 using Student's t test.

S10


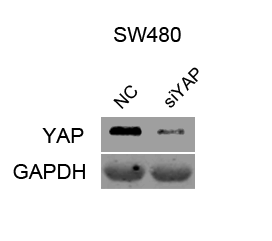


Figure S10: The interference efficiency of siYAP was checked by WB. Cells were transfected with siRNA or negative control.

S11

PCR primers used in this study were as follows:

GAPDH forward: 5′-CAAGGCCAACCGCGAGAA-3′ and reverse: 5′-CCCTCGTAGATGGGCACAGT-3′;

YAP forward: 5′-ACCCTCGTTTTGCCATGAAC-3′ and reverse:

5′-TTGTTTCAACCGCAGTCTCTC-3′;

CYR61 forward: 5′-AGCCTCGCATCCTATACAACC-3′ and reverse:

5′-TTCTTTCACAAGGCGGCACTC-3′;

The sequences of siRNAs used in this study were as follows:

Negative control: 5′-UUCUCCGAACGUGUCACGUTT-3′

siMEK1: 5′-AAGCAACUCAUGGUUCAUGCUTT-3′

siMEK2: 5′-AAGAAGGAGAGCCUCACAGCA-3′

siIQGAP1: 5′-UUAUCGCCCAGA AACAUCUUGUUGG-3′

siIQGAP1#:5′-AGUAUAUCCUGGUAAGUGGAUGC-3′

siKRAS: 5′-GCCUUGACGAUACAGCUAATT-3′

siKRAS#: 5′-GGUAGUUGGAGCUGGUGGCGUAG-3′

siβ-TrCP: 5′-GTGGAATTTGTGGAACATCTT-3′

siYAP: 5′-GACAUCUUCUGGUCAGAGA-3′

siLATS1: 5′-CACGGCAAGAUAGCAUGGA-3′

siLATS2: 5′-AAAGGCGUAUGGCGAGUAG-3′
